# Possible Interactions between the Biosynthetic Pathways of Indole Glucosinolate and Auxin

**DOI:** 10.3389/fpls.2017.02131

**Published:** 2017-12-14

**Authors:** Siva K. Malka, Youfa Cheng

**Affiliations:** ^1^Key Laboratory of Plant Molecular Physiology, Institute of Botany, Chinese Academy of Sciences, Beijing, China; ^2^School of Life Sciences, University of Chinese Academy of Sciences, Beijing, China

**Keywords:** glucosinolate, auxin, metabolism, development, Arabidopsis

## Abstract

Glucosinolates (GLS) are a group of plant secondary metabolites mainly found in Cruciferous plants, share a core structure consisting of a β-thioglucose moiety and a sulfonated oxime, but differ by a variable side chain derived from one of the several amino acids. These compounds are hydrolyzed upon cell damage by thioglucosidase (myrosinase), and the resulting degradation products are toxic to many pathogens and herbivores. Human beings use these compounds as flavor compounds, anti-carcinogens, and bio-pesticides. GLS metabolism is complexly linked to auxin homeostasis. Indole GLS contributes to auxin biosynthesis via metabolic intermediates indole-3-acetaldoxime (IAOx) and indole-3-acetonitrile (IAN). IAOx is proposed to be a metabolic branch point for biosynthesis of indole GLS, IAA, and camalexin. Interruption of metabolic channeling of IAOx into indole GLS leads to high-auxin production in GLS mutants. IAN is also produced as a hydrolyzed product of indole GLS and metabolized to IAA by nitrilases. In this review, we will discuss current knowledge on involvement of GLS in auxin homeostasis.

## Introduction

Glucosinolates (GLS) are a group of secondary metabolites found almost exclusively in Brassicaceae (Agerbirk and Olsen, 2012). GLS are nitrogen and sulfur rich compounds, forming a two-component defense system (mustard oil bomb) with myrosinases against herbivores and microorganisms (Bones and Rossiter, 1996; Rask et al., 2000). Upon tissue damage by herbivores, the mustard oil bomb comes into action, where myrosinases hydrolyze GLS into different degradation products that are toxic to the enemy (Rask et al., 2000; Chen and Andreasson, 2001). Apart from plant defense, some of the GLS degradation products are physiologically significant in plant nutrition and growth regulation (Hull et al., 2000; Rask et al., 2000; Kutz et al., 2002; Grubb et al., 2004; Katz et al., 2015). GLS breakdown products are part of human consumption and health. For instance, some of the GLS metabolites give characteristic flavors to *Brassica* vegetables (cabbage, cauliflower, broccoli, etc.) and condiments (mustard, horseradish, wasabi, etc.); and some others such as sulforaphane, indole-3-carbinol and phenethyl isothiocyanate act as cancer-preventive agents (Zhang et al., 1994; Hecht, 2000; Nakajima et al., 2001; Keck and Finley, 2004; Hayes et al., 2008). Moreover, *Brassica* crops are used for crop rotation and/or biofumigation as certain GLS metabolites exhibit natural biopesticide properties (Gimsing and Kirkegaard, 2009).

Glucosinolates are evolutionarily younger and evolved from cyanogenic glucosides. Cyanogenic glucosides are widespread *in planta*, whereas GLS are restricted to the order Capparales and the genus *Drypetes* of Euphorbiaceae (Johnson et al., 2009). Cyanogenic glucosides and GLS do not coexist in plants, except for one species, *Carica papaya* that produces both phenylalanine-derived cyanogenic glucosides and GLS (Bennett et al., 1997). Evolutionary link between cyanogenic glucosides and GLS is supported by having similarities in their biosynthesis such as amino acids as precursors and CYTOCHROME P450s (CYPs) as aldoxime metabolizing enzymes (Bak et al., 1998, 2001; Hansen et al., 2001a; Naur et al., 2003).

**FIGURE 1:**
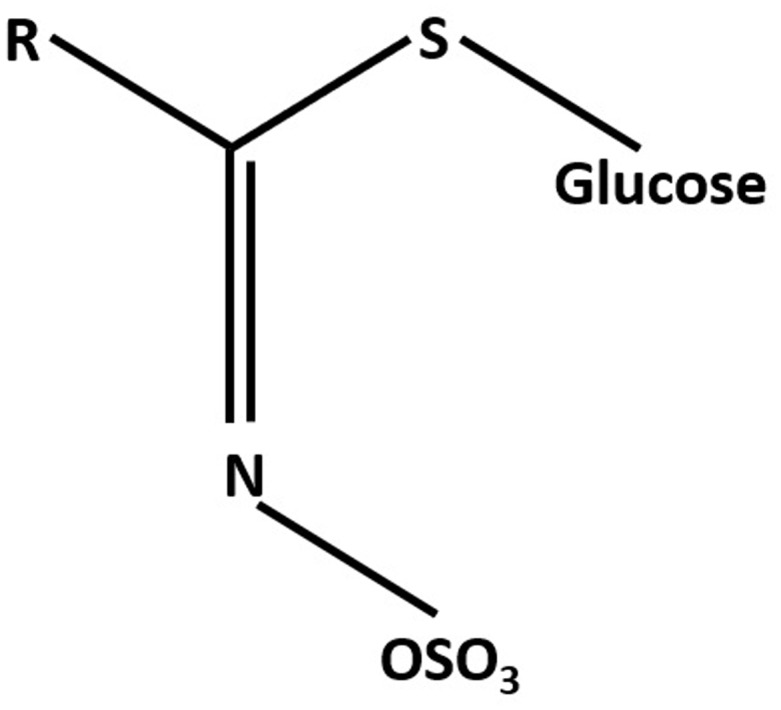
General structure of glucosinolates (GLS).

Glucosinolates (GLS) are characterized by having a thioglucose moiety, a sulfonated oxime, and a side-chain derived from aliphatic, aromatic, or indole amino acids (**Figure 1**). Currently, more than 130 different GLS structures have been identified (Agerbirk and Olsen, 2015). GLS are biosynthesized from amino acids and stored in the vacuoles of specific laticifer-like sulfur-rich cells called S-cells localized in the phloem cap along the vasculature and the leaf margins (Koroleva et al., 2010). GLS hydrolyzing myrosinases are localized in myrosin cells and are spatially separated from GLS (Thangstad et al., 1991; Xue et al., 1993; Andreasson et al., 2001; Husebye et al., 2002). Biosynthesis and long-distance transport of GLS are critical for spatio-temporal distribution of the GLS in plants (Nour-Eldin et al., 2012; Andersen and Halkier, 2014; Jørgensen et al., 2015). In Arabidopsis, transport of GLS compounds is mediated by transporter proteins GTR1/NPF2.10 and GTR2/NPF2.11 (Nour-Eldin et al., 2012).

Several lines of evidence suggest that indole GLS are metabolically linked to auxin homeostasis. Interruption of GLS metabolism leads to severe defects in plant growth and development similar to high-auxin phenotypes (Boerjan et al., 1995; Mikkelsen et al., 2000, 2004; Bak and Feyereisen, 2001; Hansen et al., 2001b; Reintanz et al., 2001; Chen et al., 2003; Tantikanjana et al., 2004; Skirycz et al., 2006; Ueda et al., 2006). The high-auxin phenotypes of GLS mutants were an effect of blocking the indole GLS pathway downstream of indole-3-acetaldoxime (IAOx), which resulted in overflow of IAOx to indole-3-acetic acid (IAA) (Halkier and Gershenzon, 2006; Nafisi et al., 2006). In this review, we discuss current knowledge on potential involvement of GLS especially indole GLS metabolism in auxin homeostasis.

## Structure and Classification of GLS

The typical chemical structure of GLS consists of β-D-glucopyranose residue linked via a sulfur atom to a (Z)-*N*-hydroximinosulfate ester plus a precursor amino acid derived R group (**Figure 1**). Based on the precursor amino acid and the types of modification to the variable R group, GLS can be classified into aromatic (phenylalanine or tyrosine), aliphatic (alanine, leucine, isoleucine, methionine, or valine), and indole GLS [tryptophan (TRP)] (Fahey et al., 2001; Agerbirk and Olsen, 2012). A list and structures of GLS can be found in an excellent review (Clarke, 2010).

## An Overview of Biosynthesis of GLS and Camalexin

**FIGURE 2:**
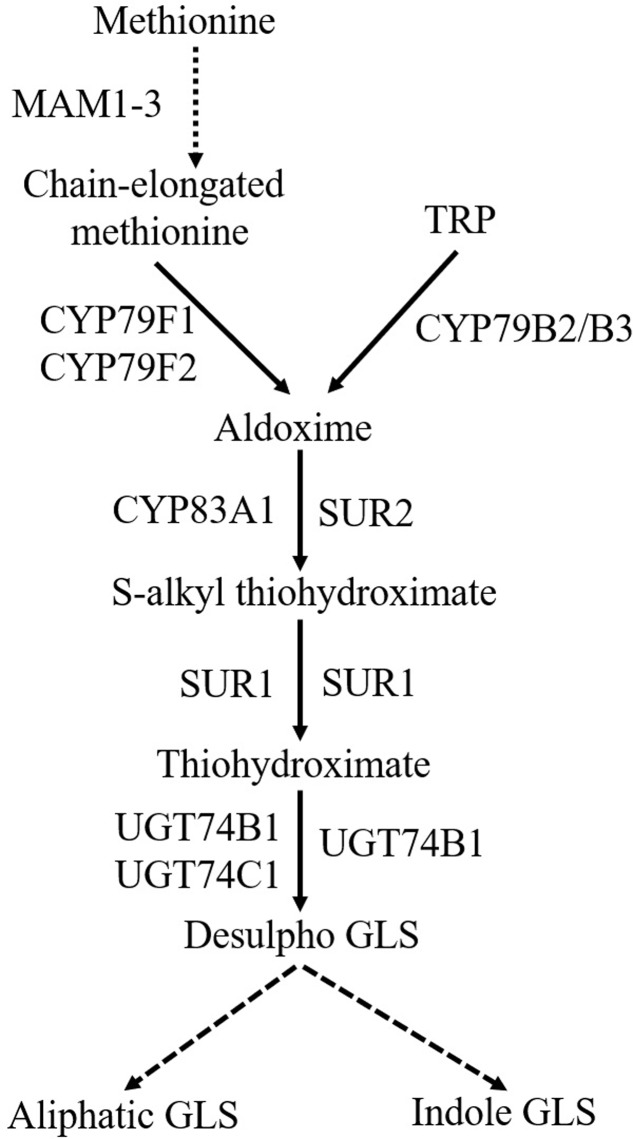
General scheme of biosynthesis of aliphatic and indole GLS. Dashed arrow denotes the process with multiple steps. MAM1-3, METHYLTHIOALKYLMALATE SYNTHASE; TRP, tryptophan; CYP79F1, CYTOCHROME P450 MONOOXYGENASE 79F1; CYP79F2, CYTOCHROME P450 MONOOXYGENASE 79F2; CYP79B2/B3, CYTOCHROME P450 MONOOXYGENASE 79B2/CYTOCHROME P450 MONOOXYGENASE 79B3; SUR2, SUPERROOT2; SUR1, SUPERROOT1; UGT74B1, *S*-GLUCOSYLTRANSFERASE 74B1; UGT74C1, *S*-GLUCOSYLTRANSFERASE 74C1; GLS, glucosinolates.

Glucosinolates biosynthetic pathway comprises three steps (**Figure 2**): amino acid chain-elongation, core structure formation, and secondary modifications (Chen and Andreasson, 2001; Halkier and Gershenzon, 2006). In the amino acid chain-elongation step, certain aliphatic and aromatic amino acids are elongated by insertion of methylene groups into their side chains. These reactions are mediated by the *METHYLTHIOALKYLMALATE SYNTHASE (MAM) 1-3* and *MAM-like (MAML)* genes (Kroymann et al., 2001; Textor et al., 2007). The amino acid moiety of either chain elongated or not, is converted to a core GLS structure in a series of reactions. During this process, amino acids are converted to their corresponding aldoximes by CYP79s (**Figure 2**). CYP79A2 catalyzes phenylalanine (Wittstock and Halkier, 2000), CYP79F1 and CYP79F2 metabolize chain-elongated methionine (Hansen et al., 2001b; Chen et al., 2003), whereas CYP79B2/B3 convert TRP (Hull et al., 2000) to their aldoximes. The enzyme catalyzing homophenylalanine is unknown. Aldoximes are metabolized to *S*-alkylthiohydroximates by CYP83s. Methionine-derived aldoximes are catalyzed by CYP83A1, whereas aromatic- and indole-acetaldoximes are catalyzed by CYP83B1/SUPERROOT2 (SUR2) (Bak and Feyereisen, 2001; Hemm et al., 2003). *S*-alkylthiohydroximates are cleaved into thiohydroximates by a C-S lyase/SUPERROOT1 (SUR1) (Mikkelsen et al., 2004), followed by *S*-GLUCOSYLTRANSFERASE (UGT) mediated glycosylation (Petersen et al., 2001). Finally, sulfonation of the desulfo-GLS is carried out by sulfotransferases (Piotrowski et al., 2004). Later, GLS core structure undergoes several secondary modifications at the side chain and glucose moiety (Hopkins et al., 2009). Side chain of aliphatic GLS is modified by oxygenation, hydroxylation, alkenylation, and benzoylation, whereas side chain of indole GLS is modified by hydroxylation and methoxylation (Sønderby et al., 2010b).

Camalexin is a major phytoalexin found in specific group of Cruciferous plants including Arabidopsis (Glawischnig, 2007; Rauhut and Glawischnig, 2009; Bednarek et al., 2011). Camalexins are synthesized in response to fungal pathogens and play positive role in their resistance (Pedras et al., 2011). In camalexin biosynthetic pathway, CYP71A13/12 convert IAOx into indole-3-acetonitrile (IAN) (Nafisi et al., 2007; Müller et al., 2015) which is then oxidized and conjugated to glutathione by glutathione-*S*-transferase GSTF6 to produce GSH (IAN) (Su et al., 2011). This GSH (IAN) is metabolized to Cys(IAN) by γ-glutamyl peptidases GGP1 and GGP3 (Geu-Flores et al., 2011) which is metabolized to camalexin by CYP71B15/PAD3 (Zhou et al., 1999; Schuhegger et al., 2006; Böttcher et al., 2009).

## An Overview of Auxin Biosynthesis

**FIGURE 3:**
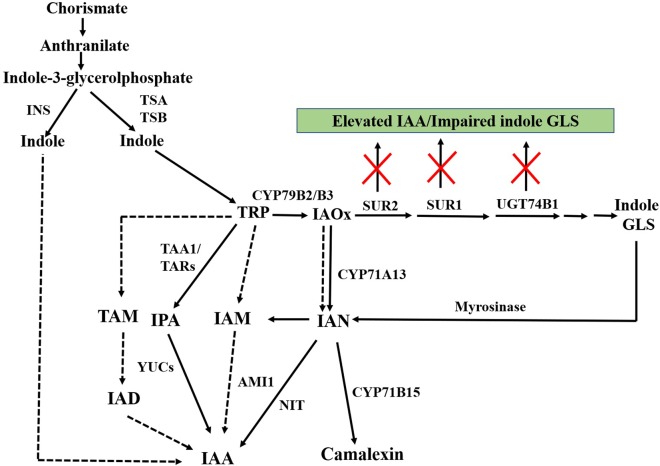
Schematic representation of TRP-independent and TRP-dependent auxin biosynthesis. IAOx metabolized from TRP acts as key metabolic branching point for biosynthesis of indole GLS, camalexin, and IAA. Inactivation of genes of post-acetaldoxime GLS biosynthesis leads to elevated IAA along with impaired indole GLS. Dashed arrows indicate pathways that are not well defined. INS, INDOLE SYNTHASE; TSA, TRP SYNTHASE A; TSB, TRP SYNTHASE B; TRP, tryptophan; CYP79B2/B3, CYTOCHROME P450 MONOOXYGENASE 79B2/CYTOCHROME P450 MONOOXYGENASE 79B3; SUR2, SUPERROOT2; SUR1, SUPERROOT1; UGT74B1, *S*-GLUCOSYLTRANSFERASE 74B1; GLS, glucosinolates; IAOx, indole-3-acetaldoxime; TAA1, TRP AMINOTRANSFERASE OF ARABIDOPSIS; IPA, indole-3-pyruvic acid; YUC, YUCCA; TAM, tryptamine; IAD, indole-3-acetaldehyde; IAM, indole-3-acetamide; AMI1, AMIDASE1; IAN, indole-3-acetonitrile; NIT, NITRILASE; CYP71B15, CYTOCHROME P450 MONOOXYGENASE 71B15; IAA, indole-3-acetic acid.

IAA is proposed to be biosynthesized from two pathways, TRP-independent and TRP-dependent pathway (Woodward and Bartel, 2005; Chandler, 2009; Normanly, 2010). TRP-dependent IAA biosynthesis is considered as the main route of IAA biosynthesis in plants (**Figure 3**).

### TRP-Independent Pathway

In the TRP-independent pathway, indole-3-glycerol phosphate or indole is the likely precursor, while the genes and enzymes involved in this pathway are still largely unknown (Ouyang et al., 2000; Zhang et al., 2008; Wang et al., 2015). Studies on TRP auxotrophic mutants that were unable to synthesize TRP revealed the existence of TRP-independent pathway (Baldi et al., 1991; Wright et al., 1991; Normanly et al., 1993; Ouyang et al., 2000; Tivendale et al., 2014). When TRP auxotrophic mutants in maize and Arabidopsis were fed with isotope-labeled anthranilate and TRP, IAA was more enriched than TRP, and the incorporation of the label into IAA from TRP was low, indicating occurrence of TRP-independent IAA biosynthesis (Wright et al., 1991; Normanly et al., 1993). The TRP biosynthetic mutants *trp3* and *trp2*, defective in TRP SYNTHASE A and TRP SYNTHASE B subunits, respectively, accumulated higher levels of IAA than the wild-type despite containing lower TRP levels (Last et al., 1991; Normanly et al., 1993; Radwanski et al., 1996; Ouyang et al., 2000). Recently, INDOLE SYNTHASE was suggested to catalyze indole-3-glyceralphosphate in TRP-independent pathway and play essential role in embryo development (Zhang et al., 2008; Wang et al., 2015).

### TRP-Dependent Pathway

In the TRP-dependent pathway, IAA is synthesized via indole-3-pyruvic acid (IPA), indole-3-acetamide (IAM), tryptamine (TAM) and/or IAOx as intermediates (Woodward and Bartel, 2005; Chandler, 2009; Normanly, 2010; **Figure 3**). The IPA pathway is considered as a predominant auxin biosynthesis pathway in plants (Mashiguchi et al., 2011; Zhao, 2014).

#### The IAM Pathway

The IAM pathway is well established in bacteria where TRP MONOOXYGENASE (iaaM) converts TRP to IAM, which is further converted to IAA by IAM HYDROLASE (iaaH) (Patten and Glick, 1996). AMIDASE 1 (AMI1) was identified as a homolog of iaaH in Arabidopsis and *Nicotiana* (Pollmann et al., 2002, 2006; Nemoto et al., 2009). However, no *iaaM* homologs have been found in plants. IAM was identified as an endogenous compound in many plant species including Arabidopsis, maize, rice, and tobacco (Sugawara et al., 2009; Mano and Nemoto, 2012; Novak et al., 2012). Additionally, AMI1 enzyme activity was detected in various plants (Kawaguchi et al., 1991, 1993; Arai et al., 2004; Pollmann et al., 2009; Sánchez-Parra et al., 2014), suggesting existence of this pathway in the plants.

#### The IPA Pathway

In the IPA pathway, the TRP aminotransferase *TAA1/TAR* (*TRP AMINOTRANSFERASE OF ARABIDOPSIS*) gene family converts TRP to IPA (Stepanova et al., 2008; Tao et al., 2008; Yamada et al., 2009; Zhou et al., 2011; Tivendale et al., 2012), which is subsequently converted to IAA by YUCCA (YUC) flavin monooxygenase (Mashiguchi et al., 2011; Stepanova et al., 2011; Won et al., 2011). Homologs of *TAA1* were identified in maize (Chourey et al., 2010; Phillips et al., 2011), pea (Tivendale et al., 2012), and several other species (Chourey et al., 2010; Liu et al., 2012). There are 11 *YUCs* in Arabidopsis, overexpression of *YUCs* results in auxin-overproduction phenotypes in Arabidopsis (Zhao et al., 2001; Woodward et al., 2005; Cheng et al., 2006; Kim et al., 2007; Hentrich et al., 2013) and various other plants (Zhao, 2014). Conversely, loss-of-function *yuc* mutants displayed low auxin synthesis with developmental defects (Cheng et al., 2006, 2007; Zhao, 2014), which can be rescued by adding auxin to growth media (Chen et al., 2014) or by expressing bacterial auxin biosynthetic gene *iaaM* under the control of a *YUC* promoter (Cheng et al., 2006). Overexpression of *TAAs* does not cause any obvious developmental phenotypes. However, low auxin phenotypes of *sav3* and *wei8* caused by mutations in *TAA1* were rescued by overexpressing *iaaM* or by using the synthetic auxin picloram (Stepanova et al., 2008; Tao et al., 2008).

#### The TAM Pathway

In the TAM pathway, *TRP DECARBOXYLASE (TDC)* converts TRP to TAM. Moreover, *TDCs* were functionally characterized to participate in indole alkaloid and serotonin biosynthesis. For instance, transgenic tobacco plants overexpressing the *TDC* gene of *Catharanthus roseus* accumulated very high levels of TAM, whereas IAA levels were unaffected (Songstad et al., 1990). Hence, the TAM pathway is not considered as a major player of auxin biosynthesis.

#### The IAOx Pathway

Indole-3-acetaldoxime is biosynthesized from TRP by CYP79B2/B3 (Hull et al., 2000). IAOx was first isolated from *Brassica oleracea* (Kindl, 1968), later conclusively identified from extracts of *Brassica campestris* (Ludwig-Müller and Hilgenberg, 1988). Earlier studies suggested that a cytochrome P450-like activity or plasma membrane-associated peroxidases might mediate conversion of TRP to IAOx (Kindl, 1968). Arabidopsis *CYP9B2/B3* were identified in a yeast screen for cDNAs conferring resistance to 5-fluoroindole (the precursor of a toxic TRP derivative) and demonstrated their ability to specifically convert TRP to IAOx *in vitro* (Hull et al., 2000; Mikkelsen et al., 2000). YUC monooxygenase was assumed to take part in IAOx synthesis, however, endogenous IAOx levels were not significantly changed in *yuc* quadruple mutants demonstrating GLS metabolism as the main contributor of IAOx synthesis (Sugawara et al., 2009; Zhao, 2014). Endogenous IAOx has not been detected in non-GLS plants such as tomato, pea, rice, maize, or tobacco (Cooney and Nonhebel, 1991; Quittenden et al., 2009; Sugawara et al., 2009), correspondingly, *CYP79B2/B3* have only been identified in GLS plants (Bak et al., 1998; Sugawara et al., 2009), suggesting IAOx synthesis is specific to GLS plants.

It has been proposed that IAOx is channeled into biosynthesis of IAA via IAN (Hull et al., 2000; Nafisi et al., 2006; Sugawara et al., 2009). It is well demonstrated that CYP71A13 catalyzes IAOx to IAN, but this gene is mainly induced in response to pathogen infection to produce camalexin (Glawischnig, 2007). IAN levels were increased in *CYP79B2* overexpressing plants, and *cyp79B2/B3* double mutants showed reduced IAN content (Zhao et al., 2002). Metabolite feeding studies showed that IAM and IAN are likely produced from IAOx (Sugawara et al., 2009). When the IAOx-deficient *cyp79b2/b3* double mutants supplied with ^13^C_6_-labeled IAOx, ^13^C_6_ atoms were efficiently incorporated into IAM, IAN, and IAA, indicating that IAA can be produced from IAOx via IAM and IAN. In consistent with this, wild-type plants supplied with IAM and IAN showed auxin-overproduction phenotypes (Sugawara et al., 2009). When Arabidopsis *CYP79B2* or *CYP79B3* genes was ectopically expressed in tobacco, IAOx and IAN were identified as endogenous compounds in the transgenic plants together with elevated levels of IAA compared to their controls (Nonhebel et al., 2011).

In addition to being metabolized from IAOx, IAN is also produced from indole GLS by myrosinases (Halkier and Gershenzon, 2006). A tendency of IAN accumulation in accordance with turnover of glucobrassicin was observed in Arabidopsis (Müller and Weiler, 2000; Reintanz et al., 2001; Zhao et al., 2002). It has been reported that low concentration of IAN can induce a high-auxin phenotype in the Arabidopsis seedlings (Normanly et al., 1997). In maize, IAN was identified as an endogenous compound at lower magnitude than Arabidopsis, exogenously supplied IAN inhibited the root growth (Thimann, 1953; Park et al., 2003; Kriechbaumer et al., 2007). The altered auxin response in the root tips of Arabidopsis myrosinase double mutants *tgg4 tgg5* was likely due to no IAN production from indole GLS under myrosinase disruption (Fu et al., 2016). *In vivo* conversion of IAN to IAA was observed in root tissue of Arabidopsis (Müller et al., 1998).

Nitrilases (NITs) are proposed to catalyze IAN to IAA (Bartling et al., 1992; Bartel and Fink, 1994; Normanly et al., 1997; Pollmann et al., 2002). Early studies assayed NIT enzyme activities from members of plant families including Cruciferae, Gramineae, and Musaceae (Mahadevan and Thimann, 1964; Thimann and Mahadevan, 1964). Arabidopsis genome contains four *NITs* named *NIT1*–*NIT4*, categorized into *NIT4* and *NIT1*-subfamilies. The members of *NIT1*-subfamily, *NIT1-3*, were suggested to be emerged from phylogenetically older *NIT4*-subfamily by gene duplication events and subsequent neo-functionalization (Piotrowski, 2008). Transgenic plants overexpressing each of *NIT1-3* were more sensitive to exogenously supplied IAN (Schmidt et al., 1996; Dohmoto et al., 2000a,b), whereas *nit* mutants were tolerant (Normanly et al., 1997). Increased NIT activity was appeared to alter levels of IAN and free IAA in *NIT1* overexpressing transgenic Arabidopsis plants (Lehmann et al., 2017). In maize, loss-of-function of *ZmNIT2*, a homolog of *AtNIT4*, caused the mutants less sensitive to IAN with significantly lower amounts of total IAA in kernels and roots of young seedlings compared to wild-type plants (Kriechbaumer et al., 2007). NIT3 was proposed to catalyze IAN to IAA in sulfur deprived Arabidopsis roots (Kutz et al., 2002). In *Brassica* plants, development of root galls caused by *Plasmodiophora brassicae* infection appeared to be mediated by IAN-derived IAA (Grsic et al., 1999; Grsic-Rausch et al., 2000; Ishikawa et al., 2007).

Apart from several lines of supporting evidence, the role of NITs in IAA biosynthesis is still arguable (Piotrowski, 2008). It was shown that these enzymes have lesser substrate preference to IAN than the compounds including phenylpropionitrile, allylcyanide, phenylthio acetonitrile, and methylthio acetonitriles raising doubt on the role of these enzymes in IAA biosynthesis (Vorwerk et al., 2001). The substrate preference of NITs would be different if the enzymes were challenged with IAN as a predominant substrate (Pollmann et al., 2002). For instance, in the roots of sulfur-deprived plants, expression of *NIT3* was strongly induced in response to intensified turnover of IAN precursor glucobrassicin and was suggested to metabolize IAA (Kutz et al., 2002). Additionally, *in planta* NITs were shown to much more efficient than the recombinant ones. For example, Arabidopsis *NIT2* ectopically overexpressed in tobacco was able to convert IAN supplied at micromolar concentrations, 15-fold below the enzyme’s apparent *in vitro K*_m_ (Schmidt et al., 1996). In the absence of exogenous IAN, neither the *nit* mutants nor *NIT* overexpressors showed severe auxin phenotypes, and endogenous IAN and IAA levels were not clearly distinguishable (Normanly et al., 1997). *NIT1* overexpressors displayed strong reduction in their primary root length with clearly elevated levels of free IAA and IAN, while *nit1-3* mutant displayed wild-type like roots with reduced total IAA levels (Lehmann et al., 2017). The *cyp79b2/b3* mutants that are deficient in glucobrassicin have strongly reduced levels of IAN (Zhao et al., 2002), however, are not affected in infection rates of *P. brassicae* and consequent root gall development (Siemens et al., 2008). The role of *NITs* in the development of clubroot was also questioned, as *cyp79b2/b3* mutants with low levels of IAN showed normal clubroot symptoms (Siemens et al., 2008). The involvement of indole GLS and *NITs* in the development of clubroot disease is seemingly more complicated. For instance, the transcripts of *BnCYP83B1* and *BnNit4* were up-regulated in the infected root of *Brassica napus* (Xu et al., 2016), whereas *CYP83B1* and other GLS biosynthesis genes *CYP79B2/B3* and *CYP83A1* were differentially downregulated during different stages of infection in *Brassica macrocarpa* (Zhang et al., 2016). Hence, further studies are needed to understand this phenomenon. The role of IAN as a direct metabolite of IAOx and the involvement of *NITs* in IAA biosynthesis was argued, because the high-auxin phenotype of *sur2* is not rescued in the *nit1* genetic background (Bak et al., 2001). The mutant impaired with all *NITs* would exclude the possible redundancy of NIT activity and gives conclusive idea on the contribution of NITs in IAA biosynthesis.

## Regulation of GLS Biosynthesis

### Transcriptional Control of GLS Biosynthesis

Various Transcription Factors (TFs) are known to regulate GLS biosynthesis at transcriptional level. Of which, some TFs have been shown to control GLS production at global level. For instance, IQD1 positively affected production of both aliphatic and indole GLS. The gain- and loss-of-function of *IQD1* led to increased and decreased accumulation of GLS, respectively (Levy et al., 2005). SLIM1, an EIN3-like TF, involved in sulfur deficiency response, was shown to repress the expression of GLS biosynthetic genes (Maruyama-Nakashita et al., 2006; Frerigmann and Gigolashvili, 2014). AtDOF1.1 was reported to promote GLS production (Skirycz et al., 2006). Finally, TFL2, an Arabidopsis homolog of HETEROCHROMATIN PROTEIN1, was shown to affect GLS biosynthesis (Kim et al., 2004; Bennett et al., 2005).

*R2R3-MYBs* constitute the largest *MYB* gene family in plants, characterized by possessing two repeats of DNA binding domains named R2 and R3 at the N-terminal end, and an activation or repression domain usually located at the C-terminus (Stracke et al., 2001). These TFs involve in various processes including development, metabolism and stress response (Chezem and Clay, 2016). Members of sub-group 12 R2R3-MYB are specific regulators of GLS biosynthesis: *MYB34*, *MYB51*, and *MYB122* control indole GLS production, whereas *MYB28*, *MYB29*, and *MYB76* regulate aliphatic GLS biosynthesis (Gigolashvili et al., 2009).

*MYB28* is characterized as a dominant regulator of aliphatic GLS, whereas *MYB29* and *MYB76* are suggested to play additional accessory role. Overexpression of these *MYBs* has been shown to induce aliphatic GLS biosynthetic genes and aliphatic GLS content (Gigolashvili et al., 2007b, 2008; Hirai et al., 2007; Sønderby et al., 2007). Consistently, loss-of-function of *MYB28* affected production of both short- and long-chain derived aliphatic GLS. However, *myb29* and *myb76* were defective in accumulation of only short-chain derived aliphatic GLS albeit to a lesser extent in *myb76* (Hirai et al., 2007; Sønderby et al., 2007; Beekwilder et al., 2008; Gigolashvili et al., 2008), indicating dominance of *MYB28* over other two *MYBs* in controlling aliphatic GLS production. In line with this, expression of aliphatic GLS biosynthetic genes was greatly affected in *myb28* than in *myb29* (Gigolashvili et al., 2007b, 2008; Hirai et al., 2007; Sønderby et al., 2007). Additionally, disruption of both *MYB28* and *MYB29* showed complete reduction of aliphatic GLS levels in *myb28 myb29* double mutants (Sønderby et al., 2007; Beekwilder et al., 2008). This shows that *MYB28* controls both short- and long-chain derived aliphatic GLS, while *MYB29* and *MYB76* regulate only the short-chain derived aliphatic GLS. However, Sønderby et al. (2010a) showed that *MYB29* and *MYB76* were able to regulate the production of long-chain derived aliphatic GLS in the genetic backgrounds of *myb28 myb76* and *myb28 myb29*, respectively, suggesting interplay of *MYBs* in controlling GLS biosynthesis. Aliphatic biosynthetic genes were differentially transactivated by these *MYBs*, though *MYB28* showed highest transactivation potential over the two *MYBs* (Gigolashvili et al., 2007b, 2008). For instance, *MAML* was strongly transactivated by *MYB28* than *MYB29*; *CYP79F2* was greatly transactivated by *MYB28* and *MYB29* but to a less extent by *MYB76* (Gigolashvili et al., 2008). Additionally, the transcript levels of aliphatic biosynthetic genes were uncoupled from the levels of GLS metabolites in the *myb28, myb29*, and *myb76* knockouts (Sønderby et al., 2010a). These reports suggest that a complex network of *MYB28*, *MYB29*, and *MYB76* controls the aliphatic GLS biosynthesis specifically and coordinately.

*MYB34/ATR1* was initially identified as a regulator of TRP biosynthesis as it controls the expression of TRP biosynthetic gene *ASA1* (Bender and Fink, 1998). The expression of indole GLS biosynthetic genes *CYP79B2/B3* and *CYP83B1/SUR2* were altered in *myb34* mutants, and transcript levels of *MYB34* were reduced *in atr4/cyp83B1/sur2*, indicating its involvement in GLS biosynthesis (Smolen and Bender, 2002). The plants constitutively overexpressing *MYB34* accumulated 10-fold higher indole GLS compared to their control plants. Conversely, *myb34* knockouts displayed low indole GLS with reduced transcript levels of indole GLS biosynthetic genes (Celenza et al., 2005). Similar to *MYB34*, both *MYB51* and *MYB122* positively regulated indole GLS production. Metabolite analysis showed increase of indole GLS levels in the plants overexpressing *MYB51* and *MYB122*, and decrease in *myb51* and *myb122* knockouts (Gigolashvili et al., 2007a). Thus, *MYB34*, *MYB51*, and *MYB122* positively regulate indole GLS production.

Because CYP79B2/B3 can convert TRP to IAOx, and the expression of *CYP79B2/B3* is regulated by *MYB34*, *MYB51*, and *MYB122*, it is possible that production of other IAOx derived metabolites including IAA, camalexin and indole-3-carboxylic acids may also be regulated by these *MYB* genes. Indeed, it was reported that *MYB34*, *MYB51*, and *MYB122* could show a conditional and probably indirect impact on the biosynthesis of camalexin and indole-3-carbolic acids (Frerigmann et al., 2015, 2016). Moreover, elevated IAA levels were found in *MYB34*, and *MYB122* overexpression lines (Celenza et al., 2005; Gigolashvili et al., 2007a), suggesting a potential role of these TFs in auxin homeostasis.

Recently, it has been shown that *bHLH04*, *bHLH05*, and *bHLH06* genes of sub-group IIIe of *bHLH* TF family take part in GLS biosynthesis together with *R2R3-MYBs* (Schweizer et al., 2013; Frerigmann et al., 2014). *bHLH06/MYC2* was shown to negatively regulate indole GLS biosynthesis, as levels of indole GLS were increased in *bhlh06/myc2* mutants (Dombrecht et al., 2007). However, it was later shown that *bHLH06, bHLH04*, and *bHLH05* positively regulate indole GLS biosynthesis, as the triple mutants had reduced levels of indole GLS (Schweizer et al., 2013; Frerigmann et al., 2014). *bHLH06* bound directly to the G-box motif in the promoters of GLS biosynthetic genes (Schweizer et al., 2013). Moreover, MYB-bHLH interactions can play essential role in controlling GLS biosynthesis. For instance, the reduction of indole GLS levels was more pronounced in *myb51 bhlh05* plants than *bhlh05* single mutants. In line with this, double gain-of-function mutants *myb34-1D bhlh05D94* had 20-fold more indole GLSs than their single mutants and wild-type plants (Frerigmann et al., 2014). These findings suggest that MYB and bHLH TFs play critical roles in regulating indole GLS biosynthesis.

### Hormonal Control of GLS Biosynthesis

Glucosinolates biosynthesis is regulated by various phytohormones, including jasmonic acid (JA), ethylene, salicylic acid (SA), and brassinosteroids (BRs) (Mikkelsen et al., 2003; Guo et al., 2013). JA is a well characterized stress signaling molecule known to integrate plant response to various environmental cues, and is involved in a wide variety of plant processes (Turner et al., 2002; Koo and Howe, 2009). JA and its precursors and derivatives collectively called as jasmonates (JAs). They are shown to induce various TFs that are involved in secondary metabolite production (Memelink et al., 2001). JAs signaling involves perception of JAs by F-box protein CORONATINE INSENSITIVE1 (COI1) protein of Skp–Cullin–F-box protein complex (SCFCOI1) that facilitates ubiquitin-26S proteasome mediated degradation of transcriptional repressors called JAZ (Jasmonate ZIM domain). JAZ proteins interact with and repress a variety of TFs; this repression is released upon JAZ degradation via JAs signaling. As JAZ proteins are known to repress sub-group IIIe of bHLH TFs, to inhibit interaction between MYB-bHLH (Chini et al., 2007), these proteins play critical role in controlling GLS biosynthesis in a JAs dependent manner. It was found that the interaction strength between bHLH-MYB proteins could affect the interaction between bHLH-JAZ proteins. An amino acid substitution in bHLH05 weakens its interaction with JAZ protein (Frerigmann et al., 2014) allowing the bHLH05 to induce indole GLS biosynthesis. Exogenous JAs treatment induced GLS biosynthetic genes and GLS content in various plant species (Brader et al., 2001; Gigolashvili et al., 2008; De Geyter et al., 2012). Therefore, MYB-bHLH interactions may play a crucial role in JAs responsive GLS biosynthesis.

Salicylic acid differentially regulates GLS biosynthesis. SA treatment has been shown to induce nearly all types of GLS, of which, 2-phenylethyl GLS showed highest accumulation in oilseed rape (Kiddle et al., 1994). In Arabidopsis, 4-methoxy-glucobrassicin was reported to be induced by SA, while the contents of glucobrassicin and neoglucobrassicin were decreased (Mikkelsen et al., 2003). Increased SA production in *mpk4* and *cpr1* induced 50% more GLS accumulation in the mutants compared to wild-type plants (Mikkelsen et al., 2003).

Glucosinolates production is negatively regulated by BR. Microarray analysis of BR-responsive genes showed that *CYP79B2* was down-regulated by BR treatment in Arabidopsis (Goda et al., 2002). BR treatment reduced accumulation of both aliphatic and indole GLS. The inhibitory role of BR was confirmed by significantly higher accumulation of GLS content in BR-deficient mutant *cpd*, whereas transgenic plant overexpressing BR biosynthetic gene *DWF4* showed dramatically reduced GLS levels (Guo et al., 2013). In another study, binding sites of BZR1 were identified in the promoters of *MYB34* and *MYB51* (Sun et al., 2010). Hence, it has been suggested that BR induced inhibition of GLS biosynthesis may be mediated by BR signaling TFs BZR1 and BES1 by binding directly to GLS biosynthetic genes or indirectly by interacting with MYB factors (Guo et al., 2013).

Further, ethylene has also been shown to induce the expression of GLS biosynthetic genes and their regulators (Mikkelsen et al., 2003; Frerigmann and Gigolashvili, 2014). Broccoli florets treated with ethylene were found to have higher quantities of specific indole GLS (Villarreal-Garcia et al., 2016). It was reported that abscisic acid (ABA) can also induce indole GLS biosynthesis (Frerigmann and Gigolashvili, 2014).

The distinct roles of indole GLS biosynthesis regulators *MYB34*, *MYB51*, and *MYB122* in response to the phytohormones have been reported (Frerigmann and Gigolashvili, 2014). *MYB34* was found to mediate ABA- and JA- induced indole GLS production. ABA and JA treatments strongly induced indole GLS biosynthesis in *myb51* and *myb122* but this tendency was not observed in *myb34* mutants. Ethylene/SA induced accumulation of indole GLS was highly affected in *myb51* than in *myb34* and *myb122*, indicating a potential role of *MYB51* in indole GLS synthesis in response to the treatment of these two hormones. *MYB122* has been suggested to play a minor role in ethylene/JA induced GLS biosynthesis (Frerigmann and Gigolashvili, 2014; Frerigmann, 2016).

Hormonal cross-talks in controlling GLS biosynthesis were also suggested. For example, methyl-JA induced specific indole GLS accumulation was significantly decreased in SA-overproducing mutant *cpr1* than in wild-type, indicating suppression of methyl-JA induced GLS biosynthesis by SA (Mikkelsen et al., 2003). In *Brassica rapa*, SA antagonistically affected methyl-JA induced GLS accumulation in the root regardless of the site of elicitation. Similar effect was found in the leaves when the roots were elicited, however, the effect was synergetic if the leaves were elicited (Zang et al., 2015).

## GLS Metabolism Is A Modulator of Auxin Homeostasis

Some of the GLS mutants were isolated from the genetic screens aimed to identify genes involved in auxin homeostasis in Arabidopsis. For instance, *sur1* was isolated in a screen designed to identify mutants with high-auxin phenotypes including small and epinastic cotyledons, an elongated hypocotyl, excess adventitious and lateral roots, and a reduced number of leaves (Boerjan et al., 1995). The different alleles of *sur1*, *alf1*, *rty*, and *hsl3*, which encodes a C-S lyase, identified in independent root morphology screens, also showed high-auxin related abnormal root morphology (Celenza et al., 1995; King et al., 1995; Lehman et al., 1996). Later, *sur2* and *rnt1*, loss-of-function mutants of CYP83B1, were found to display the phenotypes similar to *sur1* (Delarue et al., 1998; Winkler et al., 1998; Barlier et al., 2000; Bak et al., 2001). *UGT74B1* encodes a UDP-glucose:thiohydroximate *S*-glycosyltransferase. Insertional mutations in *UGT74B1* resulted in phenotypes reminiscent of auxin overproduction, such as epinastic cotyledons, elongated hypocotyls in light grown plants (Grubb et al., 2004).

The GLS mutants with high-auxin phenotypes were found to have altered levels of IAA along with impaired GLS content. In *sur1/rty*, free and conjugated IAA levels were over accumulated (Boerjan et al., 1995; King et al., 1995) with undetectable levels of all types of GLS (Mikkelsen et al., 2004). Similarly, the mutants of *UGT74B1* having excess free and conjugated IAA were associated with reduction in all types of GLS compared to their control plants (Grubb et al., 2004). Indole GLS production was reduced to 50% in *sur2* plants (Bak et al., 2001) compared to wild-type, whereas free IAA levels were increased at all developmental stages tested (Delarue et al., 1998). *cyp79f1*/*bushy1/sps* mutants showed extremely bushy phenotype (Reintanz et al., 2001; Tantikanjana et al., 2001). *cyp79f1* mutant showed decreased levels of short-chain derived GLS but increased levels of indole-3-ylmethyl-GLS, IAA (Reintanz et al., 2001), and cytokinin (Tantikanjana et al., 2001). Because the cytokinin responsive reporter *ARR5::uidA* and auxin responsive reporter *DR5::uidA* in the *cyp79f1* mutant showed that increased levels of cytokinin, but not auxin, correlate well with a root-specific expression pattern, the bushy phenotype might be caused by increased level of cytokinin (Tantikanjana et al., 2004). Both auxin and cytokinin can influence the hormone levels of each other. Increased cytokinin levels in *cyp79f1* might induce accumulation of auxin. Alternatively, increased indole GLS production in *cyp79f1* likely increased IAA biosynthesis (Mikkelsen et al., 2003). *CYP79B2/B3* were up-regulated in stressed plants, resulting in increased indole GLS and IAA (Mikkelsen et al., 2003). Therefore, it is also possible that *sps/cyp79f1* mutants were stressed because of the perturbation of cytokinin homeostasis, which in turn up-regulates *CYP79B2/B3* genes (Tantikanjana et al., 2004).

The altered IAA levels found in the GLS mutants were proposed to be synthesized from IAOx (Hull et al., 2000), a common precursor for indole GLS, camalexin, and IAA (**Figure 3**). The *CYP71* clade genes *SUR2/CYP83B1* and *CYP71A13/12* channel IAOx into biosynthesis of indole GLS and camalexin, respectively. *SUR2* catalyzes IAOx into an indole-3-*S*-alkyl-thiohydroximate and is subsequently metabolized to indole GLS (Bak et al., 2001).

Indole-3-acetaldoxime channeling into production of either IAA, or secondary metabolites indole GLS or camalexin must be tightly controlled. In *CYP79B2* overexpressing plants, elevated IAOx has been found to be channeled into biosynthesis of indole GLS (Mikkelsen et al., 2000) and IAA (Zhao et al., 2002). In response to the increased production of IAA in *CYP79B2* overexpressors, transcripts of IAA-inducible genes including *IAA/AUX*, *SAUR*, and *GH3s* were induced (Zhao et al., 2002). Consistently, disruption of *CYP79B2/B3* abolished production of indole GLS, and affected rate of IAA biosynthesis in the mutants (Zhao et al., 2002). *CYP79B2* was shown to highly express in response to silver nitrate treatment that induces camalexin synthesis. Consistently, *cyp79B2* single and *cyp79B2/B3* double mutants were unable to synthesize camalexin under induced conditions (Glawischnig et al., 2004; Ljung et al., 2005). In the absence of pathogen attack, IAN levels were not altered in *cyp71A13* knockout mutants (Sugawara et al., 2009), indicating fine-tuned regulation of IAOx metabolic channeling into the corresponding pathways. Loss-of-function mutations of *SUR2* restricted IAOx flux into biosynthesis of indole GLS, resulting in decreased indole GLS production (Bak et al., 2001) and increased free IAA levels (Delarue et al., 1998). In agreement with the elevated free IAA levels, *sur2* disruption induced transcription of early auxin responsive genes such as *Aux/IAAs* and *GH3s* (Mikkelsen et al., 2009; Morant et al., 2010). Conversely overexpression of *SUR2* led to increased production of indole GLS (Bak et al., 2001). It was shown that TAM can competitively inhibit *SUR2* that resulted in conversion of IAOx into IAA (Bak and Feyereisen, 2001). These studies indicate that IAOx plays important roles in plant development and defense responses as a branch point for biosynthesis of indole GLS, camalexin, and IAA.

Differential activation of IAOx pathway resulted in altered auxin homeostasis in post-acetaldoxime mutants or overexpressors of *CYP79B2* (Nafisi et al., 2006). For instance, in s*ur2*, increased endogenous IAA levels were associated with up-regulation of *CYP79B2*, IAA conjugation genes such as *GH3s* (Morant et al., 2010), and subsequent accumulation of IAA catabolites such as IAA-aspartate and oxindole-3-acetic acid (Barlier et al., 2000). Consistently, IAA-leucine resistant 1-like family of amidohydrolases *ILL1* and *ILL2* which release IAA from amide conjugates were down-regulated indicating that increased IAA levels were catalyzed to irreversible conjugates in *sur2* plants (Morant et al., 2010). Similar to *sur2* plants, overexpression of *CYP79B2* induced expression of auxin responsive genes and increased accumulation of IAN (Zhao et al., 2002). It appears that impaired aliphatic GLS production indirectly affects production of IAOx derived indole GLS and IAA levels, as demonstrated by upregulation of *CYP79B2/B3* genes in *CYP79F1* co-suppressed plants (Hansen et al., 200lb), and increased accumulation of indole-3-ylmethyl-GLS, IAA in *cyp79f1* mutant (Reintanz et al., 2001). Nevertheless, the increased IAA levels in *cyp79f1* mutants may not be responsible for the bushy phenotype.

It was shown that *cyp79b2/b3* double mutants displayed wild-type like IAA levels under normal growth conditions, but showed a modest decrease of free IAA levels under high temperature (Zhao et al., 2002; Sugawara et al., 2009). Overexpression of *CYP79B2* significantly elevated levels of indole GLS and IAN but with normal IAA levels (Zhao et al., 2002). It was suggested that *CYP79B2/B3* were primarily responsible for production of secondary metabolites GLS and camalexin (Glawischnig et al., 2004). These observations question whether IAOx pathway can contribute to basal IAA production, and its role in regulating plant growth and development.

Besides this, potential involvement of IAOx pathway in certain circumstances is well documented. It has been proposed that root growth under sulfur starvation is initiated by extra IAA produced from IAN (Kutz et al., 2002). IAA produced from IAOx and IPA-pathways has been shown to involve in *PIF4* mediated hypocotyl elongation in response to high temperature (Franklin et al., 2011). The expression of *TAA1* and *CYP79B2* genes were induced in response to high temperature, however, their expression was greatly reduced in *pif4-101* mutants (Franklin et al., 2011). Similarly, IAOx and IPA pathways were shown to be hyperactive during high temperature induced microsporogenesis as demonstrated by tremendous increase of transcripts of *NIT2* and *TAA1* (Rodríguez-Sanz et al., 2015). Recently, it has been demonstrated that *miR10515* promotes IAA biosynthesis via IAOx pathway under high temperature by suppressing *SUR1* (Kong et al., 2015). Overexpression of *miR10515* partially phenocopied *sur1* phenotype with repressed *SUR1* expression and elevated IAA concentration. *NIT3* expression was strongly induced or repressed in the *miR10515* overexpressing and silenced plants, respectively (Kong et al., 2015). Low auxin phenotype of *cyp79b2/b3* double mutants appeared only in high temperature grown plants (Zhao et al., 2002). These reports suggest that IAOx pathway may provide extra auxin in response to environmental stresses.

It was reported that upregulation of the IAOx pathway can compensate defects in the IPA pathway (Stepanova et al., 2008). The elevated IAA in *sur2* plants had attenuated the meristem maintenance and lateral root formation defects of *TAA1 a*nd *TAR2* double mutants *wei8 tar2*. Additionally, dwarf phenotypes of *sur2/rty1* were alleviated in *wei8 tar2 sur2* and *wei8 tar2 rty1* genetic backgrounds suggesting an existence of a functional overlap between the IPA- and IAOx-dependent routes of auxin biosynthesis (Stepanova et al., 2008). Developmental defects in *ASA1/WEI2* mutants were suppressed by excess IAA levels accumulated in *sur1* and *sur2* mutants, respectively (Stepanova et al., 2005). These findings indicate that IAOx pathway may be operative under normal growth conditions as well; however, further studies are needed to confirm this idea. Regardless of how significant this pathway is in controlling plant growth and development, endogenous IAOx and its metabolizing enzymes *CYP79B2/B3* were found only in GLS plants. Thus, IAOx pathway is considered as a species-specific pathway (Sugawara et al., 2009).

## Conclusion

Biosynthetic pathways of indole GLS, camalexin and IAA are metabolically connected by their common metabolic intermediate IAOx. Disruption of indole GLS production leads to altered auxin homeostasis via differential activation of IAOx pathway. Physiological role of NITs in IAA biosynthesis is so far not conclusive. Our current knowledge on IAOx pathway suggests that this pathway is likely operative under special circumstances. Identification of the enzyme responsible for conversion of IAOx to IAA, and its functional characterization under normal and induced conditions would help us to better understand the role of this pathway in plant development.

## Author Contributions

All authors listed have made a substantial, direct and intellectual contribution to the work, and approved it for publication.

## Conflict of Interest Statement

The authors declare that the research was conducted in the absence of any commercial or financial relationships that could be construed as a potential conflict of interest.
